# Gastric xanthoma is correlated with early gastric cancer of previously *Helicobacter pylori*‐infected gastric mucosa

**DOI:** 10.1002/jgh3.12479

**Published:** 2020-12-14

**Authors:** Narihiro Shibukawa, Shohei Ouchi, Shuji Wakamatsu, Yuhei Wakahara, Akira Kaneko

**Affiliations:** ^1^ Department of Gastroenterology NTT West Osaka Hospital Osaka Japan; ^2^ Department of Internal Medicine Osaka Police Hospital Osaka Japan

**Keywords:** eradication, gastric cancer, gastric xanthoma, *Helicobacter pylori*, natural disappearance

## Abstract

**Background and Aim:**

Successful *Helicobacter pylori* eradication has been shown to prevent the development of gastric cancer (GC), but clinical evidence for factors that correlate with GC of previously *H. pylori*‐infected gastric mucosa (after eradication or natural disappearance) is limited. The purpose of our study was to identify these correlative factors.

**Methods:**

We retrospectively examined data from patients with previously *H. pylori*‐infected gastric mucosa. Data from 168 patients who developed early GC and underwent endoscopic submucosal dissection (Group C) and 835 patients with no history of early GC (Group NC) were compared. We extracted data on gender; age; complications from malignant disease and diabetes mellitus; American Society of Anesthesiologists (ASA) physical status classification; and endoscopic characteristics of atrophy (open type), intestinal metaplasia, and gastric xanthoma (GX). Correlations were determined with multivariate logistic regression analysis and propensity score matching.

**Results:**

A significantly higher proportion of patients had GX in Group C than in Group NC. Age, male gender, ASA physical status classification of class III or higher, complications from malignant disease, atrophy (open type), and the presence of intestinal metaplasia and GX were identified as factors that correlated independently with GC (odds ratio = 3.65; 95% confidence interval = 2.37–5.61; *P* < 0.0001). Propensity score matching demonstrated that the prevalence of GC was also significantly higher in patients who were positive for GX (37.2% *vs* 18.3%; *P* < 0.0001).

**Conclusion:**

GX was shown to correlate with early GC of previously *H. pylori*‐infected gastric mucosa.

## Graphical abstract

We compared the characteristics of 168 patients with gastric cancer (GC) of previously H. pylori‐infected gastric mucosa (after eradication or natural disappearance) to those of 835 patients without GC in order to investigate factors that correlate with early GC. Gastric xanthoma was shown to correlate with early GC.

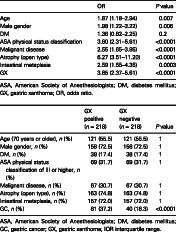

## Introduction

Gastric cancer (GC) is one of the most common causes of cancer‐related deaths worldwide.^1^ The cause of most GC is *Helicobacter pylori*, and successful *H. pylori* eradication prevents GC development.^2^, ^3^, ^4^, ^5^, ^6^ In our country, *H pylori* eradication for chronic gastritis began to be covered by the national health insurance scheme in 2013. Since then, the use of surveillance esophagogastroduodenoscopy (EGD) has increased in patients of previously *H. pylori*‐infected gastric mucosa (after eradication or natural disappearance).

Severe atrophy and intestinal metaplasia were reported as risk factors of GC after *H. pylori* eradication.^7^ We have also reported that the presence of gastric xanthoma (GX) was a useful predictive marker for early GC detected after *H. pylori* eradication.^8^ However, the factors that correlate with early GC in patients with previously *H. pylori*‐infected gastric mucosa, especially natural disappearance, remain unknown.

The present study aimed to identify these factors, including the presence of GX.

## Methods

### *Patients*

A total of 509 patients underwent endoscopic submucosal dissection (ESD) for early GC at NTT West Osaka Hospital (Osaka, Japan) between June 2006 and March 2019. Of these patients, 179 developed early GC of previously *H. pylori*‐infected gastric mucosa. Among outpatients of our hospital, 1178 previously *H. pylori*‐infected patients had no history of early GC. Patients were selected from our hospital's endoscopic information management system (NEXUS by FUJIFILM, Tokyo, Japan) and our hospital's prescription history of drugs for *H. pylori* eradication. Previously *H. pylori*‐infected gastric mucosa was defined as follows: (i) after *H. pylori* eradication and (ii) observation of endoscopic atrophy and more than two negative tests of serum antibody, the rapid urease test, immunohistochemistry, the ^13^C urea breath test, or the stool antigen test. Exclusion criteria included no atrophy at antrum, remnant stomach, endoscopic observation of the whole stomach outside of our hospital, and no EGD performed after *H. pylori* eradication at our hospital. Finally, 168 patients with GC (Group C) and 835 patients without GC (Group NC) of previously *H. pylori*‐infected gastric mucosa were enrolled. The following variables were examined: gender, age, complications from malignant disease (past history or during treatment) and diabetes mellitus (DM), past history of GC, American Society of Anesthesiologists (ASA) physical status classification, days of follow‐up, and endoscopic characteristics. Endoscopic still images were reviewed by one expert endoscopist. Endoscopic images of Group C patients were evaluated at the time of ESD or diagnosis of GC, and those of Group NC patients were evaluated at the last EGD examination.

The severity of gastric atrophy was classified according to the criteria of Kimura and Takemoto.^9^ GX was diagnosed by the presence of flat or slightly elevated yellowish‐white lesions, as revealed by conventional endoscopy. Intestinal metaplasia was confirmed in this study by the presence of a map‐like redness or a flat elevated grayish‐white lesion, as revealed by white‐light imaging without advanced diagnostic endoscopy. This study was carried out with the approval of the Ethics Committee of NTT West Osaka Hospital (Osaka, Japan). The research was conducted in accordance with the Declaration of Helsinki. Each patient provided written informed consent for ESD and EGD, after which the data were obtained anonymously. Given the nature of the data collection, the requirement for informed consent was waived for this study.

### *Statistical analyses*

All statistical analyses were performed with EZR version 1.40 (Saitama Medical Center, Jichi Medical University, Saitama, Japan), which is a graphical user interface for R (The R Foundation for Statistical Computing, Vienna, Austria). More precisely, it is a modified version of R commander designed to add statistical functions that are frequently used in biostatistics.^10^ The chi square test was performed to investigate the relationships between the two groups. Differences between the two groups were analyzed by the Mann–Whitney *U* test when the data was not parametric. Multivariate logistic regression analysis was used to identify the factors that correlate with early GC of previously *H. pylori*‐infected gastric mucosa. One‐to‐one propensity score matching was used to determine whether GX correlated with early GC in patients of previously *H. pylori*‐infected gastric mucosa. Individual propensity scores were calculated through logistic regression modeling based on the following seven covariates: age (70 years or older), male gender, complications of DM and malignant disease, ASA physical status classification class of III or higher, atrophy (open type), and the presence of intestinal metaplasia. Patients with and without GX were then paired one‐to‐one on these propensity scores using exact matching. A standard caliper width of 0.2 × log (standard deviation of the propensity score) was used. The threshold for significance was *P* < 0.05.

## Results

### *Baseline characteristics*

The baseline characteristics of the two patient groups are summarized in Table 1. Group C patients were older than Group NC patients. The proportion of male patients and patients with ASA physical status classification of III or higher were significantly higher in Group C than in Group NC. Complications with DM and malignant diseases were more prevalent in Group C patients than in Group NC patients. Fifty‐seven patients (33.9%) in Group C had past history of GC. The number of days of follow‐up in Group C was fewer than that of Group NC.

**Table 1:** Baseline characteristics of the two groups

Characteristic	Group C (*n* = 168)	Group NC (*n* = 835)	*P* value
Age, median (IQR), years	74 (68–79)	65 (59–73)	<0.0001
Male gender, *n* (%)	133 (79.2)	543 (65.0)	0.0005
DM, *n* (%)	45 (26.8)	124 (14.9)	0.0003
ASA physical status classification of III or higher, *n* (%)	103 (61.3)	192 (23.0)	<0.0001
Malignant disease, *n* (%)	87 (51.8)	165 (19.8)	<0.0001
Follow‐up, median (IQR), days	1266 (213–2429)	1459 (705.25–2827)	0.015

ASA, American Society of Anesthesiologists; DM, diabetes mellitus; GC, gastric cancer; IQR, interquartile range.

### *Endoscopic characteristics*

The endoscopic characteristics of the patients are shown in Table 2. The proportions of patients with atrophy (open type), intestinal metaplasia, and GX were significantly higher in Group C than in Group NC (58.9% *vs* 17.7%; *P* < 0.0001).

**Table 2:** Endoscopic characteristics of the two groups

Characteristic	Group C (*n* = 168)	Group NC (*n* = 835)	*P* value
Atrophy (open type), *n* (%)	152 (90.5)	347 (41.6)	<0.0001
Intestinal metaplasia, *n* (%)	141 (83.9)	422 (50.5)	<0.0001
GX, *n* (%)	99 (58.9)	148 (17.7)	<0.0001

GX, gastric xanthoma.

### *Multivariate logistic regression analysis*

Age (70 years or older), male gender, complications with DM and malignant diseases, ASA physical status classification of III or higher, atrophy (open type), and the presence of intestinal metaplasia and GX were included in the multivariate logistic regression analysis. Age (70 years or older), male gender, ASA physical status classification class of III or higher, complications from malignant disease, atrophy (open type), and the presence of intestinal metaplasia and GX were confirmed to be independent factors that correlate with GC in patients of previously *H. pylori*‐infected gastric mucosa (Table 3).

**Table 3:** Multivariate logistic regression analysis of correlated factors for gastric cancer of previously *Helicobacter pylori*‐infected gastric mucosa

	OR	*P* value
Age	1.87 (1.18–2.94)	0.007
Male gender	1.98 (1.22–3.22)	0.006
DM	1.36 (0.82–2.25)	0.2
ASA physical status classification	3.60 (2.31–5.61)	<0.0001
Malignant disease	2.55 (1.65–3.95)	<0.0001
Atrophy (open type)	6.27 (3.51–11.20)	<0.0001
Intestinal metaplasia	2.59 (1.55–4.35)	0.0003
GX	3.65 (2.37–5.61)	<0.0001

ASA, American Society of Anesthesiologists; DM, diabetes mellitus; GX, gastric xanthoma; OR, odds ratio.

### *Comparison of clinical features and endoscopic characteristics (one‐to‐one propensity score matching)*

The comparison of clinical features and endoscopic characteristics of the patients after propensity score matching is shown in Table 4. One‐to‐one propensity score matching was performed in 218 patients positive for GX and 218 patients negative for GX. The prevalence of GC was significantly higher in patients positive for GX than in patients negative for GX (37.2% *vs* 18.3%; *P* < 0.0001). C‐statistics of the propensity score matching model was 0.617 (95% confidence interval = 0.567–0.667).

**Table 4:** Comparison of clinical features and endoscopic characteristics (one‐to‐one propensity score matching)

	GX positive (*n* = 218)	GX negative (*n* = 218)	*P* value
Age (70 years or older), *n* (%)	121 (55.5)	121 (55.5)	1
Male gender, *n* (%)	158 (72.5)	158 (72.5)	1
DM, *n* (%)	38 (17.4)	38 (17.4)	1
ASA physical status classification of III or higher, *n* (%)	69 (31.7)	69 (31.7)	1
Malignant disease, *n* (%)	67 (30.7)	67 (30.7)	1
Atrophy (open type), *n* (%)	163 (74.8)	163 (74.8)	1
Intestinal metaplasia, *n* (%)	157 (72.0)	157 (72.0)	1
GC, *n* (%)	81 (37.2)	40 (18.3)	<0.0001

ASA, American Society of Anesthesiologists; DM, diabetes mellitus; GC, gastric cancer; GX, gastric xanthoma; IQR interquartile range.

### *Comparison of the location of GX and GC of previously *H. pylori*‐infected gastric mucosa*

Patients in Group C had a significantly higher prevalence of GX in all locations analyzed (Table 5). The total number of GX was not correlated with GC development. After one‐to‐one propensity score matching, the prevalence of GX at corpus, antrum and pylorus was significantly higher in patients positive for GC than in patients negative for GC (corpus 35.5% *vs* 13.3%; *P* < 0.0001, antrum and pylorus 43.8% *vs* 28.9%; *P* = 0.004).

**Table 5:** Comparison of the location of GX and GC of previously *Helicobacter pylori*‐infected gastric mucosa

Location of GX	Group C (*n* = 168)	Group NC (*n* = 835)	*P* value
Fundus, *n* (%)	17 (10.1)	37 (4.4)	0.005
Corpus, *n* (%)	58 (34.5)	46 (5.5)	<0.0001
Antrum and pylorus, *n* (%)	65 (38.7)	98 (11.7)	<0.0001

GC, gastric cancer; GX, gastric xanthoma.

## Discussion

We compared the characteristics of 168 patients with GC of previously *H. pylori*‐infected (after eradication or natural disappearance) to those of 835 patients without GC in order to investigate factors that correlate with early GC. Multivariate logistic regression analysis identified age, male gender, ASA physical status classification class of III or higher, complications from malignant disease, atrophy (open type), and the presence of intestinal metaplasia and GX as factors that independently correlate with early GC of previously *H. pylori*‐infected gastric mucosa. One‐to‐one propensity score matching revealed that the prevalence of GC was significantly higher in patients positive for GX than in those who were GX negative.

A recent retrospective cohort study in our country reported that the incidence of GC after *H. pylori* eradication was 0.35% per year.^11^ Another retrospective cohort study reported that severe atrophy and intestinal metaplasia were predictive markers for GC after *H. pylori* eradication.^7^, ^12^ However, there have been no previous studies that have investigated the factors that correlate with early GC of natural disappearance of *H. pylori*. In this study, atrophy (open type) and intestinal metaplasia were both correlated with early GC of previously *H. pylori*‐infected gastric mucosa.

GX, a localized nonneoplastic accumulation of foamy histiocytes in the lamina propria of the inflamed gastric mucosa, is occasionally observed with EGD.^13^ The presence of GX is a positive indicator of *H. pylori* infection and remains after *H. pylori* eradication therapy. Perhaps because GX was considered to be a benign entity, it has received little attention clinically.^14^ Retrospective cohort studies reported that the presence of GX was associated with the presence of GC.^14^, ^15^, ^16^, ^17^ We also reported that the presence of GX was a predictor for metachronous and synchronous GC and GC detected after *H. pylori* eradication.^8^, ^18^ However, these studies did not investigate the presence of GX as a factor that correlates with GC of natural disappearance of *H. pylori*. In this study, the presence of GX, especially in the corpus, was shown to be a correlative factor for early GC of previously *H. pylori*‐infected gastric mucosa, and the prevalence of GC was significantly higher in patients who were positive for GX. To our knowledge, this is the first study to confirm the presence of GX as a factor that independently correlates with early GC of previously *H. pylori*‐infected gastric mucosa.

Severe atrophy was present in more patients with GX,^19^ and the presence of GX has been reported to be an alerting endoscopic marker for advanced atrophic gastritis and intestinal metaplasia.^20^ This might be a potential explanation for the fact that GC develops more frequently in patients with GX.

This study has several limitations. First, it used a retrospective single‐center study design and relatively small sample sizes. Second, there was no evaluation of interobserver variability in the assessment of endoscopic findings. Finally, the study population included only patients with early GC who had undergone ESD.

In conclusion, we demonstrated that age, male gender, ASA physical status classification class of III or higher, complication with malignant disease, atrophy (open type), and the presence of intestinal metaplasia and GX were factors that independently correlated with early GC of previously *H. pylori* ‐infected gastric mucosa. These findings, especially the presence of GX, suggested improvement in the timely detection and treatment of early GC of previously *H. pylori*‐infected patients. However, further investigations are needed to confirm the utility of these factors.
